# Comparison of the Phase Transition and Degradation of Methylene Blue of TiO_2_, TiO_2_/Montmorillonite Mixture and TiO_2_/Montmorillonite Composite

**DOI:** 10.3389/fchem.2019.00538

**Published:** 2019-08-06

**Authors:** Li Zeng, Hongjuan Sun, Tongjiang Peng, Xia Lv

**Affiliations:** ^1^Key Laboratory of Solid Waste Treatment and Resource Recycle, Southwest University of Science and Technology, Ministry of Education, Mianyang, China; ^2^School of Architecture and Civil Engineering, Chengdu University, Chengdu, China

**Keywords:** TiO_2_, Ti–O–Si chemical bond, inhibition effect, the structure of montmorillonite, phase transition, absorption

## Abstract

Nano-TiO_2_ (T), TiO_2_/montmorillonite mixture (Mix), and TiO_2_/montmorillonite composite (Com) were prepared by using TiOSO_4_•2H_2_O as the precursor of TiO_2_ and montmorillonite as the matrix. The phase transition process of TiO_2_ and the degradation of methylene blue (MB) in T, Mix, and Com were studied by x-ray diffraction (XRD), infrared spectrum (IR), scanning electron microscopy with energy spectrum (SEM-EDS), and other methods. The results show that, except for the fact that the heating temperature has a great influence on the phase transition and grain growth of TiO_2_, the introduction of montmorillonite has an obvious inhibition effect on the phase transition and grain growth of TiO_2_, and the inhibition effect of the Com is obviously stronger than Mix. In Com, Ti–O–Si chemical bond was formed between TiO_2_ and oxygen atoms with negative charge on the bottom of the structure layer of montmorillonite, which is the main reason for inhibition effect. However, in Mix, TiO_2_ only covers the surface of montmorillonite without breaking the degree of order of montmorillonite and forming no chemical bond with montmorillonite, so the inhibition effect is small. From degradation of MB, it was found that before the structure of montmorillonite was destroyed (400–600°C), the total degradation percentage in Mix (85.3–99.5%) was higher than T and Com. At high temperature (above 700°C), because of the inhibition effect, the total degradation percentage of MB in Com is much larger than T and Mix, even above 1,100°C, the total degradation percentage can still reach at 47%. Therefore, in industrial applications, Mix and Com can be selected to degradation MB, according to the actual application temperature range.

## Introduction

With the rapid development of society and the fast growth of economy, the discharge of industrial and dye stuff production into wastewater is increasing, making the water pollution problem more and more serious. The problem of water pollution can be solved by using the environment-friendly technology, namely photocatalytic degradation technology, which is widely carried out currently (Abdennouri et al., 2016; Ling et al., 2019; Sun et al., 2019). As a photocatalyst with high efficiency, non-toxic, stability, high catalytic activity, and strong oxidation ability, TiO_2_ has antibacterial and bactericidal functions and can effectively degrade organic pollutants in water. It is a photocatalyst with the most potential and has a broad application prospect (Klaysri et al., 2015; Li D. et al., 2015; Calia et al., 2017; Shi et al., 2018; Kim et al., 2019). However, problems such as agglomeration, deactivation and difficulties in separation and recovery exist in the use of nano-TiO_2_, which is not conducive to the regeneration and recycling of photocatalyst and seriously affects the industrial application of TiO_2_-based photocatalytic technology (Pellegrino et al., 2017).

Montmorillonite is a common layered aluminosilicate mineral with large specific surface area and specific volume, strong adsorption capacity and stable chemical properties. It is often used as a catalyst carrier (Kadwa et al., 2014; Mofrad et al., 2018; Rao et al., 2018). The studies found that montmorillonite impregnated with TiO_2_ can maintain the photocatalytic performance of TiO_2_; at the same time the adsorption of pollutants by montmorillonite could increase the contact between TiO_2_ and pollutants and promote the photocatalysis (Rossetto et al., 2010; Dou et al., 2011; Yuan et al., 2011; Hassani et al., 2017; Liang et al., 2017). Montmorillonite particles can also cause light scattering and improve photocatalytic efficiency (Kang et al., 2010). Moreover, TiO_2_/montmorillonite composite is powder; it has a large contact area with waste liquid, high mass transfer efficiency, and good sedimentation performance, so the general sedimentation process can be used for solid–liquid separation and recycling (Djellabi et al., 2014; Zhang et al., 2015). Therefore, the composite of TiO_2_ and montmorillonite can not only solve the problem of the agglomeration and recycling of TiO_2_, but also improve the photocatalytic efficiency. When TiO_2_ enters into the interlayer of montmorillonite, montmorillonite has obvious blocking effect on the crystal phase transition and grain size of TiO_2_ (Kameshima et al., 2009; Hassani et al., 2017; Huo et al., 2018). Even at high temperature, TiO_2_ can still maintain anatase phase with good photocatalytic activity, which is beneficial to broaden the range of TiO_2_ photocatalytic application (Yuan et al., 2006; Chen et al., 2012, 2013). However, when TiO_2_ intercalation enters into montmorillonite, the degree of order of montmorillonite will be reduced (Yuan et al., 2006; Kameshima et al., 2009), which has a certain impact on the adsorption performance of montmorillonite. In a certain range, the organic pollutants, adsorbed on the montmorillonite in water, will be reduced, so as to reduce the degradation ability of organic pollutants. Therefore, whether TiO_2_ and montmorillonite are combined or not, whether TiO_2_ enters into the interlayer of montmorillonite or not, the degradation ability of organic pollutants is different.

Based on this, nano-TiO_2_ was prepared by uniform precipitation method with titanyl sulfate as precursor of TiO_2_. TiO_2_/montmorillonite composite was prepared by hydrolyzation–intercalation method with montmorillonite as substrate. The TiO_2_/montmorillonite mixture was prepared by grinding and mixing nano-TiO_2_ with montmorillonite directly. The phase transition of TiO_2_ at different temperature and the degradation of methylene blue under ultraviolet light of these three samples were studied.

## Experimental Procedure

### Raw Materials and Reagents

**Figure 1:**
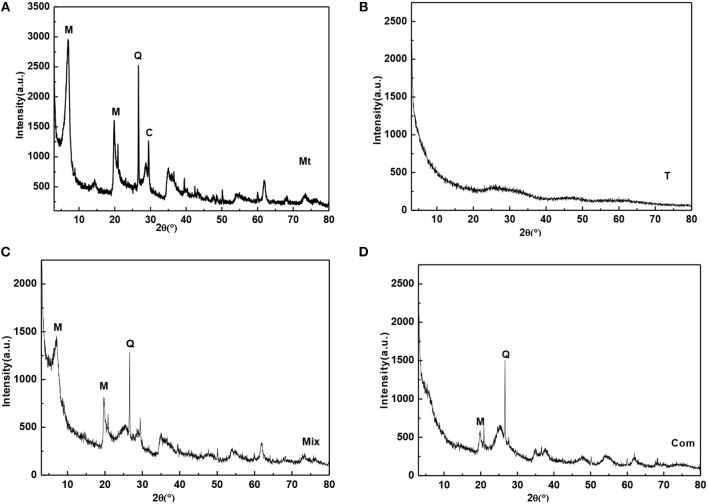
XRD patterns of **(A)** Mt, **(B)** T, **(C)** Mix, and **(D)** Com (M-montmorillonite; Q-quartz; C-calcite).

Montmorillonite was from the bentonite deposit in Santai County, Sichuan Province. The sample was purified by sedimentation method and then natrified by sodium carbonate, which was labeled as Mt. As can be seen from Figure 1A, Mt contains a small amount of quartz (d_101_ = 3.34 Å) and calcite (d_104_ = 3.03 Å). During the natrification of montmorillonite, calcium ions in the interlayer domain are exchanged by sodium ions. The calcite is formed from the reaction of Ca^2+^ with ${\text{CO}}_{3}^{2-}$. The chemical composition of Mt is SiO_2_ 59.77%, Al_2_O_3_ 15.05%, Fe_2_O_3_ 3.22%, CaO 2.84%, MgO 3.72%, Na_2_O 0.17%, and K_2_O 0.85%. The loss of ignition (LOI) and the cation exchange capacity (CEC) of Mt is 14.4% and 119.52 mmol/100 g, respectively. Mt was calcined in furnace for 2 h at 400°C to 1,400°C, and the obtained samples were labeled as Mt-400, Mt-500…, and Mt-1100, based on the calcining temperature.

The titanyl sulfate (Tianjin Guangfu Fine Chemical Research Institute, China), ammonium hydroxide (Chengdu Kelong Chemical Co Ltd, China), sulfuric acid (Chengdu Kelong Chemical Co Ltd, China), and methylene blue (Xilong Scientific Co Ltd, China), used in the experiment, were all analytically pure.

### Preparation of TiO_2_ Nano-Powder

Titanyl sulfate solution (200 ml; concentration of 0.754 mol/L) was placed in a beaker, then ammonium hydroxide with a concentration of 13% was slowly trickled into the beaker under magnetic agitation until the pH value of the solution was 4.0 (which was higher than the pH of complete precipitation of titanium hydroxide); then titanium oxide hydrate gel was obtained by filtration and washing until no ${\text{SO}}_{4}^{2-}$ was detected with BaCl_2_. After drying the titanium oxide hydrate gel at 80°C for 6 h in the oven, TiO_2_ nano-powder was obtained, which was labeled as T (Figure 1B). It can be seen that T is amorphous. T was calcined in furnace at 200°C to 1,200°C for 2 h, started to crystallize and underwent a phase transition. The obtained samples were labeled as T-t, where t was the calcining temperature.

### Preparation of TiO_2_/Montmorillonite Mixture

T (0.0275 g) was mixed with 0.0225 g Mt for mechanical grinding and homogenization; the mechanical mixture sample of TiO_2_/montmorillonite was labeled as Mix (Figure 1C). Known from the figure, montmorillonite was not intercalated, and the basal reflection was not changed compared with Mt (Figure 1A). Mix was calcined in furnace for 2 h from 200°C to 1,200°C, and the obtained samples were labeled as Mix-200, Mix-300, …, and Mix-1200 based on the calcining temperature.

### Preparation of TiO_2_/Montmorillonite Composite

Mt (1 g) was added into 100 ml ultrapure water in a beaker and stirred for 2 h to form montmorillonite suspension. Sulfuric acid solution (40%) was used to adjust the pH value of the montmorillonite suspension to 0.5, and then 12.5 mmol titanyl sulfate solution was slowly dropped into the suspension with stirring for 0.5 h to obtain the polymeric titanium ion/montmorillonite suspension. Then, 13% ammonium hydroxide was dropped into the polymeric titanium ion/montmorillonite suspension at a speed of 20 d/min, until the pH was 4.0 to obtain the hydrated titanium oxide/montmorillonite slurry. After aging for 8 h, the slurry was filtered and washed several times with ultrapure water until no ${\text{SO}}_{4}^{2-}$ was detected with BaCl_2_. At last, the hydrated titanium oxide/montmorillonite compound (Com) was obtained after drying the filter residue for 6 h under 80°C. The XRD pattern of Com (Figure 1D) showed that the d_001_ diffraction peak of Mt disappeared, indicating the sample was intercalated montmorillonite, the polymeric titanium ion entered into the interlayer of montmorillonite through cation exchange, and the layered structure of montmorillonite was destroyed by the hydrolysis of the polymeric titanium ion to form hydrated titanium oxide. Com was roasted in furnace at 300°C to 1,200°C for 2 h and the obtained samples were labeled as Com-t, where t was the roasting temperature.

### Adsorption and Photocatalysis Experiments

The MB solution with a concentration of 20 mg/L was prepared and 100 ml was measured into a 150-ml beaker. Then, 0.0275 g T-t series samples was weighed and added into the beaker under dark conditions with stirring for 4 h at room temperature (25°C). So did the 0.0225 g Mt-t series samples, 0.05 g Mix-t series samples, and 0.05 g Com-t series samples. After 4 h of adsorption treatment, the sample was irradiated with ultraviolet light (λ = 320 nm) for 0, 20, 40, 60, 120, and 180 min, respectively. Ultraviolet light is supplied by 350 W NG250Z2 high pressure sodium lamp. The sample after irradiated with ultraviolet light was centrifuged to remove the particulate matter. The residual concentration of MB was measured by the UV-3150 spectrophotometer (λmax = 660 nm).

### Characterization

The phase composition of samples was determined by X-ray diffraction (XRD, X Pert pro, Cu Ka radiation under the operating conditions of 40 kV and 40 mA, and step size 0.02°, 2θ range 3°-80°, scan speed 0.4°/S).The microstructure and elemental analysis of sample were observed using SEM/EDS, Ultra 55, operating at 15 kV at magnifying multiple ranges of 5,000. Nicolet 5700 infrared absorption spectrometer for FTIR analysis, scanning range: 4,000–500 cm^−1^.

## Results and Discussion

### Phase Transformation

**Figure 2:**
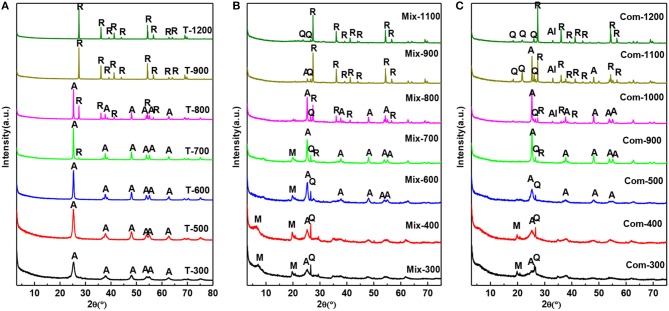
XRD patterns of the **(A)** T, **(B)** Mix, and **(C)** Com calcinated at the different temperatures (M, montmorillonite; Q, quartz; A, anatase; R, rutile; Al, Al_2_TiO_5_).

It can be seen from Figure 2 that the roast temperature and the addition of montmorillonite have a great influence on the phase transition of TiO_2_. From Figure 2A the crystallization of anatase is relatively complete at 300°C. As the roast temperature has increased to 700°C, the diffraction peak of anatase becomes sharper and the characteristic (110) of rutile (2θ = 27.42) begins to appear (Jing et al., 2003). At 900°C, the diffraction peak of anatase disappears completely, meaning anatase has been completely transformed into rutile phase. Compared with Figure 2A, because of the TiO_2_ mechanically mixed with montmorillonite, the temperature of the complete crystallization of anatase rises to 400°C and the temperature, at which anatase completely converts to rutile, has been increased to 1,000°C. In Figure 2C, the XRD pattern of Com-300 and Com-400 only appears the characteristic (020) of montmorillonite, meanwhile disappearance (001) of montmorillonite compared with Figure 2B. This indicated that the hydrated titanium oxide has been uniformly inserted into the interlayer of montmorillonite and the superimposed and disordered hydrated titanium oxide/montmorillonite compound has been formed. With the roast temperature, increasing from 500 to 900°C, the diffraction peak of anatase becomes sharp and characteristic peak of rutile begins to appear at 900°C. At 1,100°C, there is still a weak diffraction peak of anatase. By 1,200°C, anatase has been completely transformed into rutile phase.

By comparison, it was found that the transition temperature of TiO_2_ in Com from anatase to rutile appeared at 900°C and complete at 1,200°C, which was higher than that of Mix and T. The results show that the structure layer of montmorillonite has obvious blocking effect on the transition from anatase to rutile phase (Zhu et al., 2002), and the retarding effect of the Com is stronger than that of Mix. It is speculated that TiO_2_ particles in the Mix engaged with the surface of montmorillonite layer, so there is a certain blocking effect. While in Com, TiO_2_ particles not only cover the surface of montmorillonite, but also enter into the interlayer of montmorillonite and form a connection with the structural layer of montmorillonite, so the blocking effect is very obvious. Interestingly, a new phase, Al_2_TiO_5_ (Huo et al., 2018), was generated above 1,000°C in Com, which was not appeared in the high temperature process of T and Mix. Therefore, it can be determined that in the Com, TiO_2_ interacts with the montmorillonite surface layer.

### The Relative Content and Average Particle Size of TiO_2_

The crystal size (D) of TiO_2_ (anatase and rutile) in T-t, Mix-t, and Com-t series samples can be calculated by using Scherrer equation (Sakai et al., 2004; Sun et al., 2015).

The relative content of rutile phase in TiO_2_(W_R_) can be calculated by the following formula (Spurr, 1957):

$$\begin{array}{l} {\text{W}}_{\text{R}}=\frac{1}{1+0.8\frac{{\text{I}}_{\text{A}}}{{\text{I}}_{\text{R}}}}\times 100\% \end{array}$$

I_A_ and I_R_ are the intensity of the diffraction peak of the anatase (101) andrutile (110), respectively.

**Figure 3:**
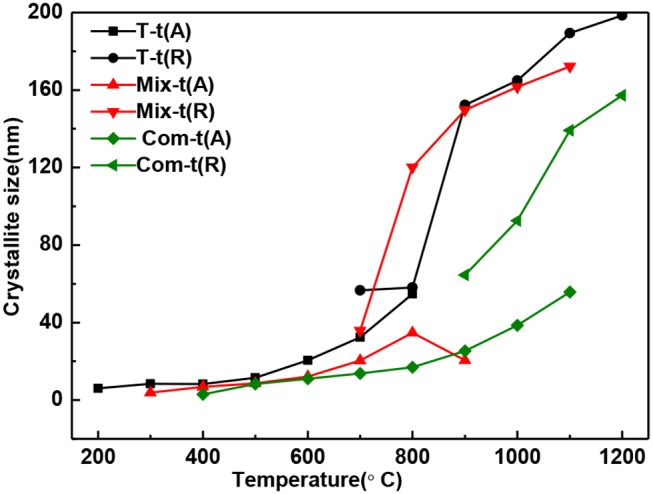
The grain size change of anatase and rutile in T, Mix, and Com calcinated at the different temperatures.

In general, the grain size of anatase and rutile increases with the roasting temperature (Figure 3). When treating at the same temperature, the grain size of anatase and rutile in Com series samples are smaller than that of Mix and T series samples. It means that the structure layer of montmorillonite also has an obvious inhibition effect on the growth of TiO_2_ grains (Kameshima et al., 2009; Huo et al., 2018), and the inhibition effect of the Com is greater than that of Mix.

**Figure 4:**
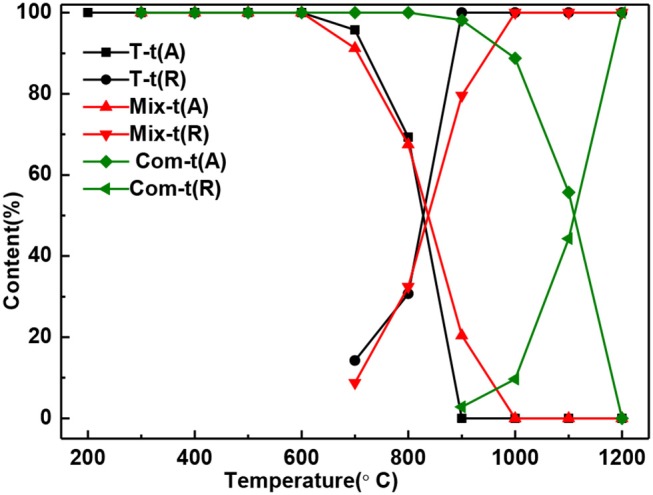
The content of anatase and rutile in T, Mix, and Com calcinated at the different temperatures.

With the increase of temperature, the relative content of anatase decreases and rutile increases (Figure 4). The relative content of anatase is reduced to 0% at 900°C in T, 1,000°C in Mix, and 1,200°C in Com, respectively, which is consistent with the results of Figure 2. Combined with Figures 3, 4, montmorillonite not only has prominent inhibition effect on the phase transition of TiO_2_ but also has obvious inhibition effect on the growth of anatase and rutile grains, and the inhibition effect of the Com is the strongest. It is believed that this is related to the link between TiO_2_ and oxygen atoms from the bottom of the montmorillonite structure in Com, just as the connection between attapulgite and TiO_2_ in TiO_2_/Attapulgite composite (Li X. Z. et al., 2015). On the one hand, the Si–O–Ti chemical bond is formed, that is, the Ti at the edge of TiO_2_ particles is combined with the oxygen at the bottom of the montmorillonite structure. On the other hand, the montmorillonite structure layers on both sides have a limited effect on TiO_2_ particles, which has delayed the transition of TiO_2_.

### Comparison of Degradation of MB

**Figure 5:**
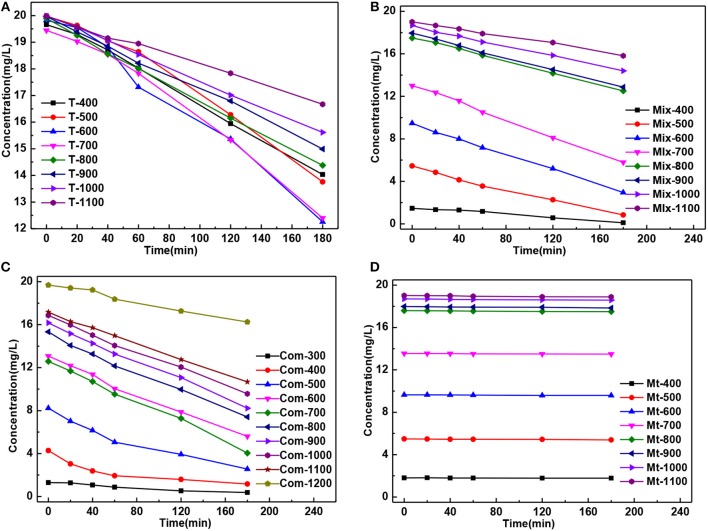
The curve of MB residual concentration of solution degradation by the **(A)** T, **(B)** Mix, **(C)** Com, and **(D)** Mt calcinated at the different temperatures vs. the time of action.

The residual concentration of MB solution degradation by the T series samples presented “fan-shaped” change, while “trapezoidal” change by the Mix, Com and Mt series samples, which was caused by smaller specific surface area of T series samples (Figure 5).

Montmorillonite has a high absorption to MB (Kang et al., 2018; Wang et al., 2018), so when discussing the photocatalytic degradation percentage of Mix and Com series samples, adsorption degradation percentage, caused by Montmorillonite, must be considered. The degradation, caused by photocatalysis, is called photocatalytic degradation percentage. The degradation percentage was calculated using the following equation from the MB residual concentration curve of different samples after the adsorption and photocatalytic treatment process in Figure 5.

$$\begin{array}{l} {\text{R}}_{\text{t}-\text{s}}=\frac{C_{0}-{\text{C}}_{\text{t}-\text{s}}}{C_{0}}\times 100\% \end{array}$$

The degradation percentage of MB solution was R_t−s_ (Xu et al., 2011), when s = 0, the adsorption degradation percentage of the samples to MB is R_t−0_, when s = 3 h, the total degradation percentage of the samples to MB is R_t−3_, so the photocatalytic degradation percentage R_t_ of the sample was R_t_ = Rt_−3h_–R_t−0_.

**Figure 6:**
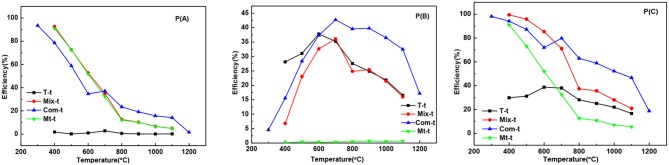
The curves of the adsorption degradation percentage **P(A)**, the photocatalytic degradation percentage **P(B)**, and the total degradation percentage **P(C)** of T-t, Mix-t, Com-t, Mt-t.

The adsorption degradation percentage of T series samples to MB was about 1% at 400°C to 1,100°C [Figure 6P(A)] showing that with the increase of temperature, the growth and phase transition of TiO_2_ grain had little influence on adsorption degradation rate of MB. The adsorption degradation percentage of Mix, Com, and Mt series samples were obviously higher than that of T series samples. It means that the adsorption degradation percentage of MB is mainly caused by the montmorillonite and the grain growth and phase transition of TiO_2_ have little influence on the adsorption degradation percentage. When temperature ranges from 400 to 600°C, the adsorption degradation percentage of Mix samples was higher than that of Com while after high temperature treatment (700–1,100°C) showed an opposite trend. The main reasons are as follows: hydrated titanium oxide just contacts with the surface of montmorillonite, but not induced into the interlayer space of montmorillonite and not destroy the original layered structure of montmorillonite in Mix samples. When roasted below 600°C, the original specific surface area of montmorillonite is retained. Therefore, Mix-400, Mix-500, and Mix-600 have a higher adsorption degradation percentage. With the increase of temperature (700 to 1,100°C), the structure of montmorillonite is easier to damage, so adsorption degradation percentage reduced and lower than Com. However, hydrated titanium oxide entered into the interlayer of montmorillonite, distributed evenly both in the surface and interlayer of montmorillonite, destroyed the original adsorption of montmorillonite in Com samples. So, Com-400, Com-500, and Com-600 had relatively lower adsorption decolorization percentage. Rising the temperature, dehydration of hydrated titanium oxide in the interlayer of montmorillonite, the specific surface area of montmorillonite decreased relatively slowly and the adsorption degradation percentage was relatively high. In addition, the inhibition effect of Com on the phase transition and grain growth of TiO_2_ is stronger than that of the Mix, so the specific surface area of the Com drops lower and its adsorption percentage is higher at high temperature (700 to 1,100°C).

It can be seen from the Figure 6P(B), the photocatalytic degradation percentage of T, Mix, and Com samples increases first and then decreases, which is closely related to the change in the crystal phase and grain size of TiO_2_. While there is no TiO_2_ in the Mt-t series samples, there is no photocatalytic degradation percentage. At 400–600°C, the photocatalytic degradation percentage of T series samples was higher than Mix and Com, as the anatase phase, produced by T, is more complete. With the increase in temperature, the anatase is more easily transformed to rutile in T than that of Mix and Com, hence, the photocatalytic degradation percentage of T samples is lower. Com samples have higher photocatalytic degradation percentage than Mix samples roasted at the same temperature; because, in Com, the inhibition effect of montmorillonite layer on the phase transition and grain size of TiO_2_ is greater than in Mix. At 700–900°C, Com series samples have relatively high photocatalytic degradation percentage, especially at 700°C. The particle size of anatase increases with the increase in temperature (800°C), so the specific surface area decreases and photocatalytic degradation percentage decreases slightly. At 900°C, anatase (3.2 ev) begins to transform into rutile (3.0 ev) and a close contact rutile phase thin layer is formed on the surface of anatase grains. As the anatase and rutile phase have different Fermi levels (Spurr, 1957), Schottky potential barrier is generated between the interfaces, which promotes the separation, transfer, and migration of internal electron-hole pairs to the surface of TiO_2_ (Gao et al., 2001), and promotes the photocatalytic degradation percentage of MB. When the temperature is increased, the content of anatase phase is less, the grain size is bigger, and the migration distance of electron-hole pairs to the surface of the particle is longer, so the photocatalytic degradation percentage is reduced obviously, but still high.

The overall trend of the total degradation percentage of MB by T series samples [Figure 6P(C)] was similar to that of the photocatalytic degradation percentage [Figure 6P(B)], and in the same sample, the photocatalytic degradation percentage was significantly higher than the adsorption degradation percentage. Therefore, the degradation of T series samples to MB solution was mainly caused by the photocatalytic degradation. While the total degradation percentage of Mt series samples to MB solution was only caused by the adsorption degradation. The total degradation percentage of Mix and Com series samples to MB solution are the combination of adsorption and photocatalytic degradation percentage. When the temperature is lower than 600°C (the temperature before the destruction of the montmorillonite structure), adsorption plays a major role, while higher than 600°C, photocatalysis plays a major role. This is also related to the inhibition effect of the phase transition and grain growth produced by the TiO_2_ entering into the interlayer of montmorillonite. In general, the total degradation percentage of Com and Mix to MB is higher than that of T. and total degradation percentage of Mix to MB is higher than Com before the structural damage of montmorillonite, that is, before 700°C. Therefore, in practical applications, the choice of Mix or Com can be made according to the applied temperature. For fields requiring degradation at high temperatures (>700°C), such as ceramic coating, Com can be chosen. Mix can be selected for degradation below 700°C.

### Connection Between TiO_2_ and Montmorillonite Structure Layer

**Figure 7:**
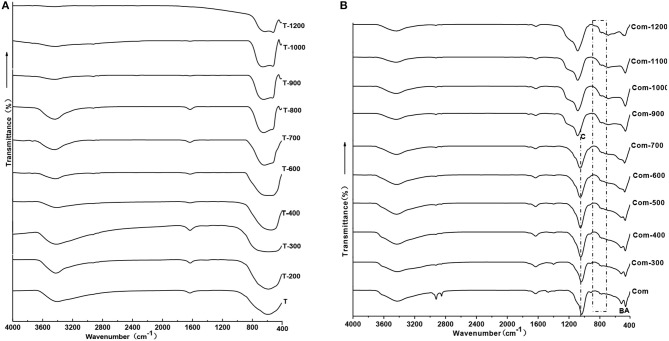
IR spectroscopy patterns of **(A)** T and **(B)** Com calcinated at different temperature.

In order to confirm the molecular vibration characteristics of Com samples and whether Ti–O–Si chemical bond is formed by the TiO_2_ and the bottom oxygen on the surface of montmorillonite structural layer, the infrared spectroscopy analysis is carried out on the T and Com series samples, the results are shown in Figure 7.

Combined with Figure 2A, below 600°C, TiO_2_ is anatase phase and there is a wide absorption band in the range is 400–900 cm^−1^ in the infrared spectrum. Above 900°C, anatase transformed to rutile, showing a broad absorption band within the range of 450–800 cm^−1^, a shoulder absorption band appears near 520 cm^−1^, and a small absorption band appears near 410 cm^−1^.With the increase of roasting temperature, the broad absorption band of the sample gradually narrowed. This indicates that the Ti–O vibration mode in rutile structure is gradually narrowed, and the crystallinity of the sample is gradually improved.

**Figure 8:**
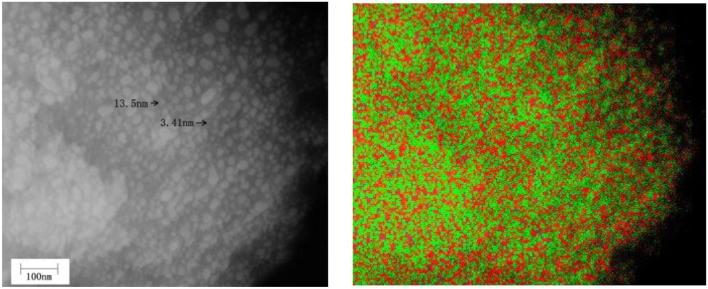
SEM image of TiO_2_/montmorillonite composite (Com-700) **(Left)** and image of Si (red)–Ti (green) composition distribution in corresponding region **(Right)**.

The infrared spectral characteristics of montmorillonite mainly show in the strong Si–O stretching vibration absorption band within the range of 900–1,100 cm^−1^, the Si–O bending vibration absorption band near 515 cm^−1^ and the Al–O vibration absorption band near 465 cm^−1^ (Tichit et al., 1988). Combined with Figure 2C, the phases below 700°C are montmorillonite and anatase, above 900°C, structure of montmorillonite layer is completely damaged, TiO_2_ is a mixture of anatase and rutile. Below 700°C, except the strong Si–O stretching vibration absorption band near 1,040 cm^−1^(C) (Abdennouri et al., 2016), the Si–O bending vibration absorption band near 515 cm^−1^(B) and Al-O vibration absorption band near 465 cm^−1^(A), there exists a weak absorption band range in 750–950 cm^−1^. Above 900°C, due to the complete destruction of montmorillonite structure, the absorption band of Si–O stretching vibration moved to 1,140 cm^−1^, the Si–O bending vibration absorption band and the Al–O vibration absorption band combined into an absorption band. Interestingly, weak absorption bands in the range of 750–950 cm^−1^ are more obvious, and the analysis suggests that these weak absorption bands may be related to the stretching vibration of Si–O–Ti (Hasegawa et al., 1991; Sosnov et al., 2010). In the infrared spectrum of Com samples, a weak wide absorption band appeared near 930 cm^−1^, which did not appear in the infrared spectrum of montmorillonite, anatase, and rutile. It was believed that the absorption band was caused by Si–O–Ti anti-symmetric stretching vibration (Hassani et al., 2015, 2017). This indicates that TiO_2_ particles formed Ti–O–Si chemical bond with oxygen at the bottom of the structure layer of montmorillonite in Com. It can also be seen from Figure 8 that TiO_2_ particles are evenly distributed on the surface and between layers of montmorillonite, and the element distribution of Si and Ti is also uniform.

In the preparation process of Com, titanyl sulfate solution was hydrolyzed into the polymeric titanium ion by controlling the pH of montmorillonite suspension to 0.5, and the polymeric titanium ion with different degree entered into the interlayer of montmorillonite through cation exchange. With the increase of pH in the solution, the polymeric titanium ion was further hydrolyzed, and its hydrolysis and the resulting hydrated titanium oxide molecules could be expressed by Equation (1). Continuous polymerization of hydrated titanium oxide molecules can form a molecular network of titanium oxide, which can be expressed by Equation (2).


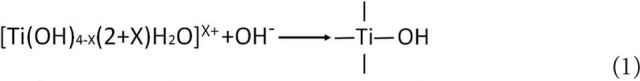



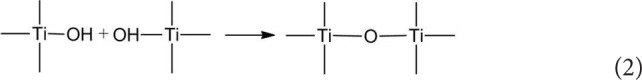


The hydrated titanium oxide molecules, formed by Equation (1) or the titanium oxide molecules, formed by Equation (2), can form chemical bonds with the negatively charged oxygen atoms at the bottom of the montmorillonite structure, as shown in Equation (3) and Equation (4).


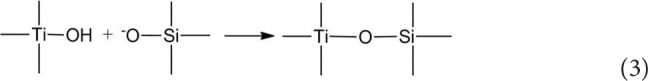



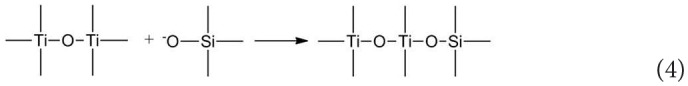


Therefore, in the interlayer of montmorillonite or on the surface of the layer of montmorillonite, TiO_2_ can form a chemical bond with the bottom oxygen on the bottom layer of the montmorillonite structure, that is, Ti-O-Si chemical bond. Hence, the Ti–O–Si absorption band in the infrared spectrum of Com samples can be theoretically explained. In other words, in Com samples, anatase or rutile nanoparticles bond with silica skeleton [Si_4_O_10_]_n_ in the structure layer of montmorillonite, which give rise to the inhibition of the phase transition of TiO_2_ particles and grain growth.

## Conclusion

1. Anatase was completely transformed into rutile phase in T at 900°C. Because of the addition of montmorillonite, the temperatures, at which anatase was completely converted to rutile phase in the Mix and Com, were increased to 1,000 and 1,200°C, respectively. The addition of montmorillonite also had an obvious inhibition effect on the growth of TiO_2_ (anatase and rutile) grains. After treatment at the same temperature, the anatase grains and rutile grains of T series samples and Mix series samples were 4–34 and 15–88 nm larger than Com series samples, respectively.
2. Effect of the degradation of methylene blue is closely related to the heat treatment temperature, the crystal structure of TiO_2_ and grain size. When treated at low temperature (400 to 600°C), as the structure of montmorillonite is not destroyed and the adsorption capacity is kept, the degradation rate of MB is highest in Mix. While at high temperature (700 to 1,100°C), as the Ti–O–Si chemical bond was formed with TiO_2_ particles and oxygen at the bottom of the structure layer of montmorillonite in Com, it had stronger degradation ability.
3. In practical application, the choice of Mix or Com can be made according to the application temperature of the degradation of MB. Mix can be selected in the low temperature range (the temperature before the destruction of the montmorillonite structure), while Com in the high temperature range.

## Data Availability

All datasets generated for this study are included in the manuscript.

## Author Contributions

HS designed the research. XL and LZ conceived the experiments and analyzed the results with the help of HS and TP. LZ drafted the manuscript.

### Conflict of Interest Statement

The authors declare that the research was conducted in the absence of any commercial or financial relationships that could be construed as a potential conflict of interest.
